# Microscopic Characteristics and Controlling Factors of Pore Structures in Shale Reservoirs: A Case Study of the Chang 7 Member in the Longdong Area of the Ordos Basin

**DOI:** 10.1021/acsomega.5c08201

**Published:** 2025-10-31

**Authors:** Guan Li, Wenxiang He, Zhigang Wen, Yong Hu, Xiaoyang Gao, Na Xiao

**Affiliations:** † College of Resources and Environment, 47897 Yangtze University, Wuhan 430100, China; ‡ College of Petroleum Engineering, Yangtze University, Wuhan 430100, China; § Hubei Key Laboratory of Oil and Gas Drilling and Production Engineering, Yangtze University, Wuhan 430100, China

## Abstract

The Ordos Basin is renowned for its development of continental and transitional marine-continental shale formations, particularly within the Triassic Yanchang Formation’s Chang 7 member, which functions as a primary stratum for shale oil reservoirs. These reservoirs are characterized by pronounced heterogeneity, posing substantial challenges to their exploitation. In order to facilitate the assessment and potential evaluation of these shale oil reservoirs during exploratory phases, the present study concentrates on the Lower Triassic Chang 7 shale located in the southwestern region of the Ordos Basin. Employing a diverse array of analytical techniques—such as X-ray diffraction (XRD), total organic carbon (TOC) measurement, nitrogen adsorption (N_2_), high-pressure mercury intrusion (HPMI), microscopic thin sections, cast thin sections, and argon ion polished scanning electron microscopy—this research meticulously examines the reservoir spaces within silty shales. The lithological composition is predominantly composed of siliceous minerals, with the reservoir spaces largely consisting of mineral matrix pores that include both intergranular and intragranular porosity. The distribution of pore volume is primarily occupied by macropores and mesopores, while the structure of these pores is significantly influenced by the TOC content and the prevalence of siliceous minerals. The volumetric presence and specific surface area of macropores and mesopores, alongside pore radius, demonstrate a negative correlation with TOC levels and a robust positive correlation with felsic content, although they exhibit a pronounced negative correlation with clay minerals. Conversely, the correlation with carbonate rock minerals appears to be negligible. Collectively, the pore structure is influenced by the content of organic matter, felsic, and clay minerals, with felsic exerting the most substantial impact. The research results indicate that pore development in the Chang 7 Member shale reservoirs of the Longdong area is jointly controlled by mineral composition and the degree of OM evolution. This suggests that, in practical exploration, siliceous-rich, clay-poor silty shales generally offer more favorable reservoir conditions. Conversely, intervals with relatively high OM content that have not yet reached the hydrocarbon generation threshold require integrated evaluation alongside their thermal evolution stage to more accurately assess their reservoir potential.

## Graphical abstract

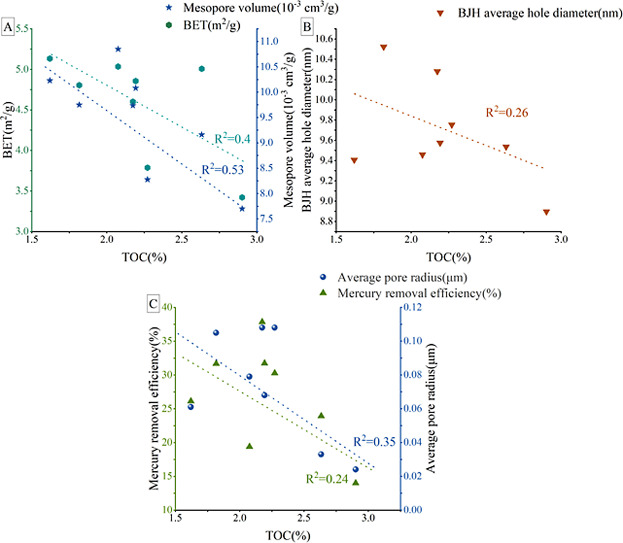

## 1 Introduction

As global energy demands escalate and advancements in technology progress, nontraditional oil and gas reservoirs—encompassing tight sandstones, coalbed methane, and shale oil and gas—have become focal points for exploration and development worldwide. China has seen considerable advancements in the field of shale oil and gas exploration and production, with substantial shale oil reserves being discovered in basins including the Bohai Bay, Songliao, Junggar, and Ordos.^1^ The majority of China’s terrestrial shale formations are lacustrine in nature. Under the fast-paced alterations in depositional facies and the complicated geological backgrounds, these lacustrine shales exhibit greater heterogeneity.^2^ In lacustrine environments, sediment supply and hydrodynamic conditions affect the shale’s organic content, granularity, and thickness, thus influencing porosity formation and distribution.^3^ A minority of lacustrine shales are characterized predominantly by organic pores, while the majority possess intergranular and intragranular porosity.^4,5^ Thus, studying the pore structure and influential factors of lacustrine shale oil reservoirs holds significant importance.

In the past few decades, research on reservoir testing methods has made considerable progress, providing excellent opportunities for studying the pore systems in shale. High-pressure mercury intrusion (HPMI) and low-pressure nitrogen adsorption (N_2_) techniques can characterize the shale’s pore structure and quantitatively measure its architecture.^6–10^ Observational techniques such as argon ion SEM and cast thin sections enable the capture of pore morphology characteristics and spatial distribution patterns, as well as the associations with minerals and organic matter (OM), on a microscopic scale, thereby providing visual support for detailed characterization of pore structures.^11–13^ Techniques like Fourier transform infrared microscopy (FTIR), nuclear magnetic resonance (NMR), and infrared spectroscopy based on atomic force microscopy (AFM-IR) offer new perspectives in microscopic characterization of pores;^14–18^ microcomputed tomography (micro-CT) provides a nondestructive, high-resolution imaging perspective.^19,20^ These techniques have been extensively utilized in studying the internal structures of organic-rich shales.^21–24^ There is extensive research on the factors of shale porosity characteristics, with the core determinants primarily centered around the content of organic carbon, thermal evolution extent, and mineral composition. In this context, the process of hydrocarbon generation from organic carbon is closely associated with the development of porosity, manifesting as a rise in the count of organic pores proportionate to the amount of generated hydrocarbons.^25^ Regarding the influence exerted by thermal maturity on porosity, the pattern is more complex; the characteristics of porosity initially increase with maturity, subsequently decrease, and eventually increase again, illustrating a nonlinear evolutionary trend.^26^ In terms of the effects exerted by mineral composition on shale porosity characteristics, the role of clay minerals (CMs) in the development of micropores is particularly significant, displaying a positive connection between their content and the volume of micropores.^27^ The influence exerted by different minerals on pore size varies, specifically, a rise in quartz content promotes macropore development, while CMs exert a certain influence on the development of mesopores.^28^ In the study of the formation and storage mechanisms of shale porosity, the interaction between rigid particles and CMs represents a critical focal point. CMs, distributed around rigid particles, often undergo deformation, forming crescent-shaped nanopores.^29^ Further research indicates that the periphery of rigid particles in shale not only serves as a favorable site for pore preservation^30,31^ but also, a higher content of these particles tends to enhance micropore development.^32,33^ Beyond the interactions between rigid particles and minerals, the contribution of OM to porosity has also garnered significant attention. Investigations into the Barnett Shale have revealed that OM constitutes the primary source of its porosity;^34^ this pattern has been corroborated by regional statistics, with studies on North American shales indicating that organic material contributes over 50% to the nanopores.^35,36^

The Ordos Basin is characterized by complex shale types, largely due to the geological transition from marine to terrestrial environments during the Carboniferous to Permian periods. This transition facilitated the development of transitional shales within the Benxi Formation, Taiyuan Formation, and Shanxi Formation, encompassing an area of 250,000 km2.^37–39^ In the Triassic system of the Ordos Basin, lacustrine shales are predominantly developed, whereas marine shales in the Sichuan Basin are typically represented by the Lower Silurian Longmaxi Formation and the Lower Cambrian Niutitang Formation. These marine shales primarily feature OM pores, which are nearly circular and include intergranular pores. The pore sizes range from 10 to 250 nm, with mesopores accounting for 48.58% of the pore structure. These OM pores are well-developed, with small pores nested within larger macropores, resulting in an average specific surface area (SSA) of 36.73 m^2^/g and good connectivity. Conversely, terrestrial shales in the Ordos Basin are dominated by CM intergranular pores, with fewer intragranular pores and some OM containing microfractures. Pore sizes range from 2.3 to 5.2 nm (cylindrical) and 2.6 to 4.2 nm (plate-like/microfractured), with a predominance of mesopores and micropores. These OM pores are less prevalent and less developed, leading to a smaller SSA. Transitional shales, however, develop both intergranular and OM pores, with intergranular pores exhibiting various shapes and OM pores displaying a honeycomb structure. The pore sizes range from 1 to 60 nm, primarily between 1 and 6 nm and 40–60 nm. Although these OM pores are poorly developed, the micropores contribute to a larger SSA, averaging 62.20 m^2^/g.^4,10,28,40–42^ In the Chang 7 Member silty shales of the Ordos Basin, OM, primarily sapropelic kerogen with 2% to 6% TOC, governs the development potential, morphology, and connectivity of organic pores through threshold effects, compositional variations, and occurrence states, thereby serving as the material basis for pore formation.^43^ Thermal evolution exerts a stage-dependent influence on pores, marked by an initial increase followed by a subsequent decline. Regarding mineral composition, rigid minerals such as quartz and feldspar support pore structures and provide dissolution space, whereas CMs exhibit both constructive and destructive effects. Together with OM and thermal evolution, these factors synergistically shape the effective pore system.^44^

The heterogeneity of lacustrine shale reservoirs is pronounced, with complex pore types that are critical for understanding the characteristics of lacustrine pores and the factors influencing pore heterogeneity. Through a combination of X-ray diffraction (XRD), liquid N_2_, HPMI experiments, and microscopic analyses using thin sections, cast thin sections, and AIP-FESEM, this research explores the factors affecting the pore space in reservoirs. It investigates the control exerted by organic carbon content, siliceous minerals, carbonate rocks, and CMs on the development of pores in shale oil reservoirs. This comprehensive study provides a basis for predicting reservoir quality in similar conditions, thereby supporting shale oil exploration.

## 2 Geological Setting

The Ordos Basin is a Mesozoic sedimentary basin formed through a prolonged process of superimposed evolution.^45,46^ The basin’s geological structure is characterized by its subdued relief, lacking anticlines and faults, and exhibits a regional slope from higher elevations in the east to lower elevations in the west. Based on current structural features, it can be divided into six principal tectonic units: the Yishan Slope, the Yimeng Uplift, the Weibei Uplift, the Western Margin Thrust Belt, the Tianhuan Depression, and the Jinxi Flexure Zone (Figure 1A;^45^). Situated in the southwestern part of the Ordos Basin (Figure 1B), the Longdong region spans across the Yishan Slope and Tianhuan Depression, stretching from Wuqi in the north to Jingchuan in the south, and from Yingjiacheng in the west to Zhidan in the east.^47^ In the Longdong region, the Upper Triassic Yanchang Formation’s (YF) Chang7 member developed significant deposits of thick oil shale and thin fine sandstone layers, serving as the primary producing zones for tight oil. The Chang7 strata typically have a thickness of about 110 m, with variations between 100 and 120 m (Figure 1B;^48^). The Chang7 member is subdivided into three sublayers: Chang7_1_, 7_2_, and 7_3_. The sedimentary provenance of the Longdong area predominantly originated from the southwest. The Chang7_1–2_ sections primarily consist of semideep to deep lacustrine subfacies characterized by gravity flow and turbidite deposits, with lithologies including silty mudstone, mud-rich siltstone, and mudstone. During this period, hydrothermal activity peaked, and the proliferation of lake algae and plankton provided abundant organic material for the formation of organic-rich shales. The Chang7_3_ section features organic-rich black shales interbedded with minor tuff and carbonaceous mudstone. Overall, the Chang7 segment exhibits high organic carbon content, serving as the principal source rock for hydrocarbons in the Mesozoic reservoirs of the Ordos Basin (Figure 1B;^47–50^ ). The reservoir rocks in the area cover feldspar lithic sandstone and fine-grained lithic feldspar sandstone. The Longdong area displays porosity values (8.0%–12.0%), with an average of 9.3%, and permeability primarily distributed between 0.01 and 1.00 × 10^–3^ μm^2^, averaging 0.18 × 10^–3^ μm^2^, which categorizes it as a typical low-porosity and low-permeability shale oil reservoir.^49^

**Figure 1:**
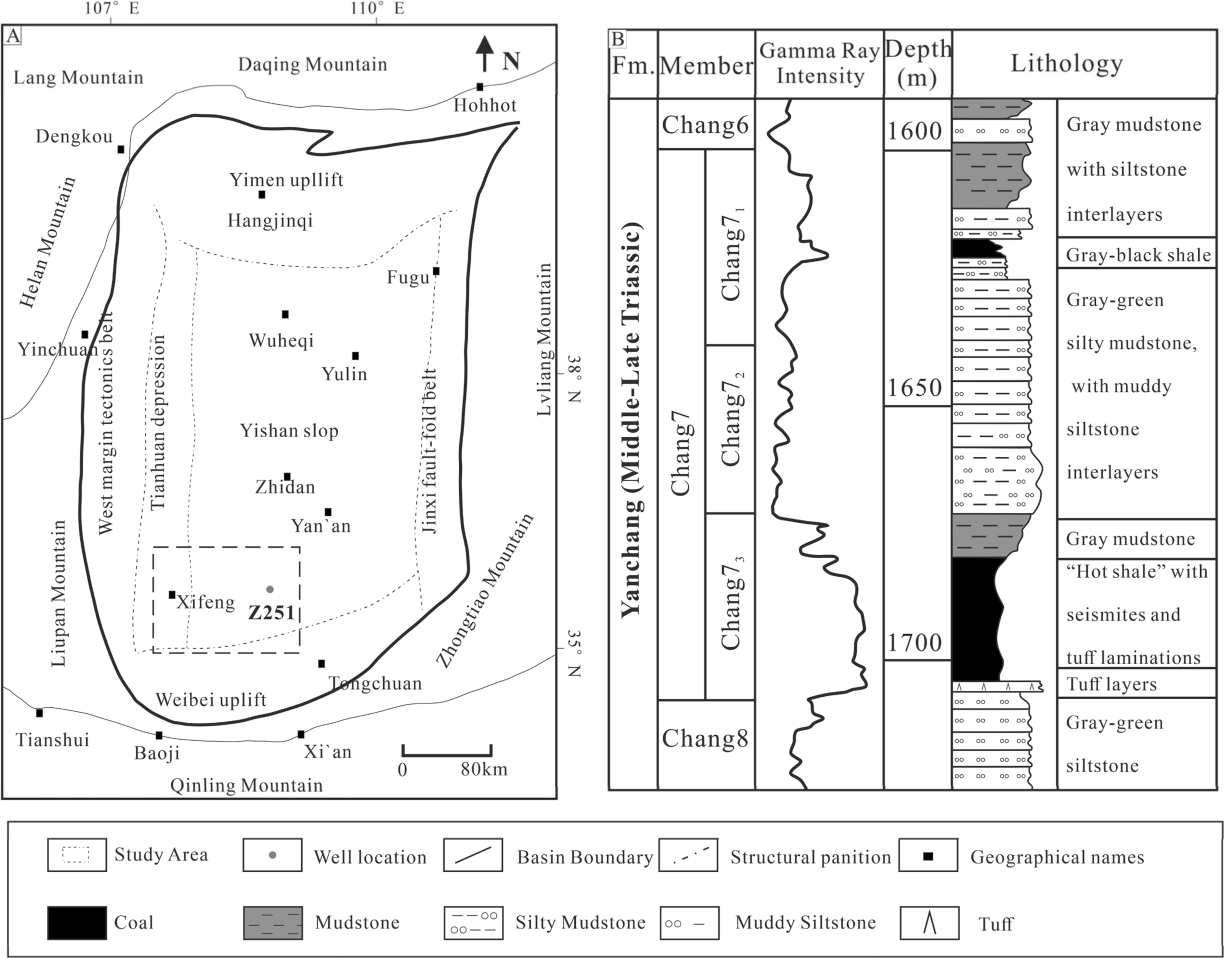
(A): tectonic division of the Ordos Basin and location of the study area (modified from^49^). (B): Stratigraphic division of the Upper Triassic YF Chang7 strata in the Longdong area, Ordos Basin.

## 3 Sample Collection and Analytical Techniques

A selection was made of a single iron pillar well in the eastern Longdong area, from which samples of silty shale were collected at eight different depths. These samples were subjected to a variety of analyses, including total organic carbon (TOC) analysis, XRD, CM analysis, HPMI, and N_2_ experiments. Additionally, the residual portions of these eight samples were prepared for microscopical examination through grinding and polishing to extinction. This preparation facilitated the creation of thin sections, cast thin sections, argon ion polishing, and examination using field emission SEM (AIP-FESEM).

### 3.1 TOC Experiment

All collected core samples were initially washed with deionized water, then dried at low temperatures, and pulverized to 120 mesh. A sample mass between 80 and 120 mg was placed into a crucible, soaked in dilute hydrochloric acid (prepared at a volumetric ratio of HCl\H_2_O = 1:7) for 24 h to fully react and remove inorganic carbon from the sample. Following this, samples were washed using deionized water until neutral, followed by drying at 80 °C to remove moisture. The TOC content was then measured using a LECO CS-230 carbon–sulfur analyzer.

### 3.2 XRD Experiment

This experiment was conducted to gather data on the mineralogical composition of the rocks. The analysis was performed using a Bruker AXS D8 Discover X-ray diffraction instrument at the Key Laboratory of Oil and Gas Resources and Exploration Technology at Yangtze University. Prior to testing, the samples were ground to 120 mesh, and part of the sample was flattened on a glass slide.^51^

### 3.3 N_2_ Experiment

Initially, 2–3 g of the sample powder was prepared and degassed at 110 °C for 12 h for moisture and impurity removal from the pore surface. The quantity of nitrogen adsorbed and desorbed was recorded at relative pressures ranging from 0.0005 to 0.99. The SSA was calculated using the BET theoretical model,^52^ and pore volume (PV) was determined using the BJH theoretical model.^53^ The instrument employed was MicroActive for ASAP 2460 2.01.

### 3.4 HPMI Experiment

Prior to testing, the samples were dried at 105 °C to constant weight. The mercury intrusion experiment involved both pressurization to introduce mercury and depressurization to withdraw it, peaking at 200 MPa. The equipment used included the Corelab CMS300 from the United States and the AutoPore IV 9500 mercury porosimeter.

### 3.5 Microtherplate Experiment

Eight samples were set as ordinary sections and tested for mineral composition using a Canon EOS 700D polarizing microscope (Canon), with further identification of mineral components in the same regions of the thin sections using a Leica polarizing microscope (DM4500P).

### 3.6 AIP-FESEM Experiment

The pretreatment of the samples was critical for observing the porosity of the shale. An Ilion+ 697C argon ion polisher (Gatan, USA) was used for polishing the samples, which measured 10 mm in width, 5 mm in height, and 1.5 mm in length. The polished surface was coated with a 0.8 nm carbon layer to enhance conductivity.^54^ Subsequently, the porosity of the shale was examined using a Quanta 450 field emission scanning electron microscope (FEI, USA).

## 4 Results

### 4.1 Mineralogical Composition and OM Content

The mineralogical composition of shale primarily reflects the depositional environment and diagenetic evolution during sedimentation and maturation processes.^40^ The mineralogy of shale can be categorized into three types: siliceous minerals (including quartz, potassium feldspar, and plagioclase), CMs, and other authigenic minerals. Consequently, the mineralogical composition of shale, as determined by XRD analysis, is summarized in Tables 1, 2 and illustrated in a ternary diagram (Figure 2). In the Longdong area’s Chang7 member of the YF, the shale is predominantly quartz-rich, with its mineral components primarily comprising quartz and feldspar, followed by CMs and carbonates. Siliceous minerals are predominantly quartz and feldspar, with quartz content ranging from 49.1 to 71.6%, and feldspar primarily consisting of potassium feldspar and plagioclase, accounting for 4–10% and 11.8–19.5% respectively. The content of calcite, dolomite, and siderite in carbonate rocks varies from 0.9% to 2.9%, 0 to 2.5%, and 0.4 to 4.4% respectively. The content of CMs ranges from 6.5 to 21.8% (Table 1), with an extremely low pyrite content of 0.5%, occurring in only one sample.

**Table 1:** Data of TOC and Rock Mineral Composition of the Chang7Member of the YF in the Triassic System

			rock mineral compositions (%)									
sample ID	depth (m)	TOC (%)	quartz	K-feldspar	plagioclase	calcite	dolomite	siderite	pyrite	ankerite	muscovite	clay
Z251-1	1612.15	2.90	49.1	4.0	15.8	0.9	2.5	4.4	/	/	1.5	21.8
Z251-2	1617.88	1.82	59.3	10.0	19.5	1.8	2.1	0.8	/	/	/	6.5
Z251-3	1630.33	2.19	65.0	4.6	16.9	1.7	/	0.4	/	2.2	/	9.2
Z251-4	1630.86	1.62	64.2	5.2	14.3	2.9	2.2	0.7	/	1.5	/	9.0
Z251-5	1636.22	2.63	71.6	4.5	11.8	2.2	/	0.5	/	1.6	/	7.8
Z251-6	1637.85	2.17	69.2	5.4	11.9	1.0	/	0.6	/	2.1	/	9.8
Z251-7	1656.11	2.27	56.2	4.7	18.1	2.2	/	0.7	/	2.5	1.0	14.6
Z251-8	1672.02	2.08	64.4	6.3	13.7	1.8	2.3	0.9	0.5	0.7	/	9.4

**Table 2:** Data of TOC and CM Composition of the Chang7Member of the YF in the Triassic System^a^

			relative content of CMs (%)				mixed layer ratio (S, %)
sample ID	depth (m)	TOC (%)	I/S	illite	kaolinite	chlorite	I/S
Z251-1	1612.15	2.90	56	31	3	10	16
Z251-2	1617.88	1.82	40	39	6	15	14
Z251-3	1630.33	2.19	60	31	2	7	15
Z251-4	1630.86	1.62	60	29	3	8	18
Z251-5	1636.22	2.63	55	30	3	12	12
Z251-6	1637.85	2.17	50	37	4	9	15
Z251-7	1656.11	2.27	53	29	5	13	14
Z251-8	1672.02	2.08	61	27	3	9	17

a Note: I/S refers to Illite/smectite mixed layer. C/S refers to chlorite/smectite mixed layer.

**Figure 2:**
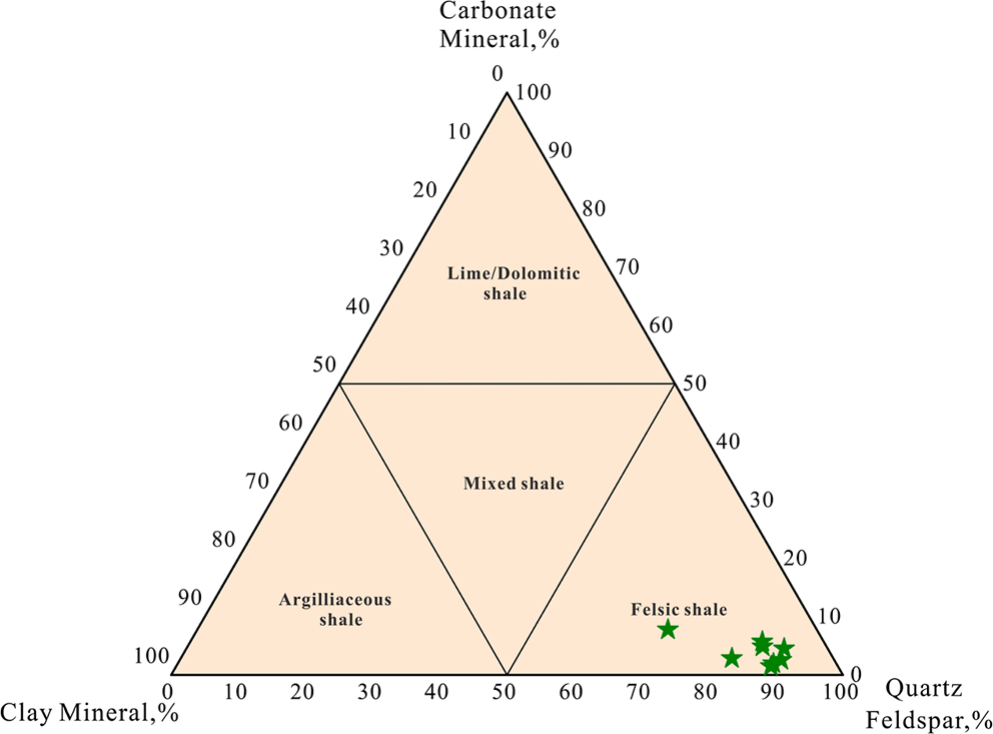
Triangular diagram of shale mineral composition (reference to board format^55^).

The CM suite includes montmorillonite, Illite, Illite/smectite (I/S) mixed layers, kaolinite, and other subcategories.^56^ XRD results indicate (Table 2) that the I/S mixed layer is the predominant CM in the study area, followed by Illite and glauconite, with the lowest content of kaolinite. The average relative contents are 53%, 34%, 9.8%, and 3.2% respectively. This is consistent with findings from several scholars indicating that the high I/S values in the terrestrial Chang7 shales suggest a low stage of diagenesis.^40,57,57^ Based on the measured data of total organic carbon (w­(TOC)), the w­(TOC) in the Chang7 segment of the Longdong area ranges from 1.62 to 2.90%, with an average of 2.22%.

### 4.2 Quantitative Analyses of Pore Structure

#### 4.2.1 N_2_ Experiment

The N_2_ experiment facilitates the acquisition of several key parameters of shale, such as SSA, total PV, and average pore diameter, as indicated in Table 3. The SSA of shale samples, calculated through the Brunauer–Emmett–Teller (BET) equation, ranged from 4.42 to 5.13 m^2^/g, with an average value of 4.58 m^2^/g. The total PV, determined using the Barrett–Joyner–Halenda (BJH) model, was between 10.9 and 15 cm^3^/kg, averaging at 13.5 cm^3^/kg. The pore size distribution, also analyzed using the BJH model, varied from 8.9 to 10.52 nm, with an average of 9.8 nm.

**Table 3:** PV and SSA Obtained by Gas Adsorption from the Sample of Z251

			pore structure parameters					
			BET (m^2^/g)	BJH (10^–3^ cm^3^/g)				BJH (nm)
sample ID	depth (m)	TOC (%)		macropore	mesopore	micropore	SUM	
Z251-1	1612.15	2.90	3.42	3.07	7.70	0.12	10.90	8.90
Z251-2	1617.88	1.82	4.80	3.69	9.75	0.24	13.68	10.52
Z251-3	1630.33	2.19	4.86	3.85	10.08	0.23	14.15	9.57
Z251-4	1630.86	1.62	5.13	4.06	10.23	0.32	14.60	9.41
Z251-5	1636.22	2.63	5.01	3.47	9.16	0.34	12.98	9.54
Z251-6	1637.85	2.17	4.60	3.70	9.74	0.28	13.72	10.28
Z251-7	1656.11	2.27	3.79	4.47	8.28	0.22	12.97	9.75
Z251-8	1672.02	2.08	5.04	3.88	10.85	0.27	15.00	9.46

According to the classification principles of the International Union of Pure and Applied Chemistry (IUPAC), the curves formed between adsorption quantities and relative pressure are referred to as isotherms.^58,59^ Analysis of the isotherms for the samples revealed a distinct hysteresis loop between the adsorption and desorption curves, characteristic of a typical Type II isotherm.^59,60^ However, the morphology of different hysteresis loops varied significantly, attributable to differences in the internal pore structures of the shale (Figure 3). Therefore, the characteristics of the hysteresis loops can be utilized to clarify pore morphology, specifically including H1, H2, H3, and H4 types, which correspond to open pores, ink-bottle pores, plate-like slit pores, and unilateral slit pores, respectively.^61^

**Figure 3:**
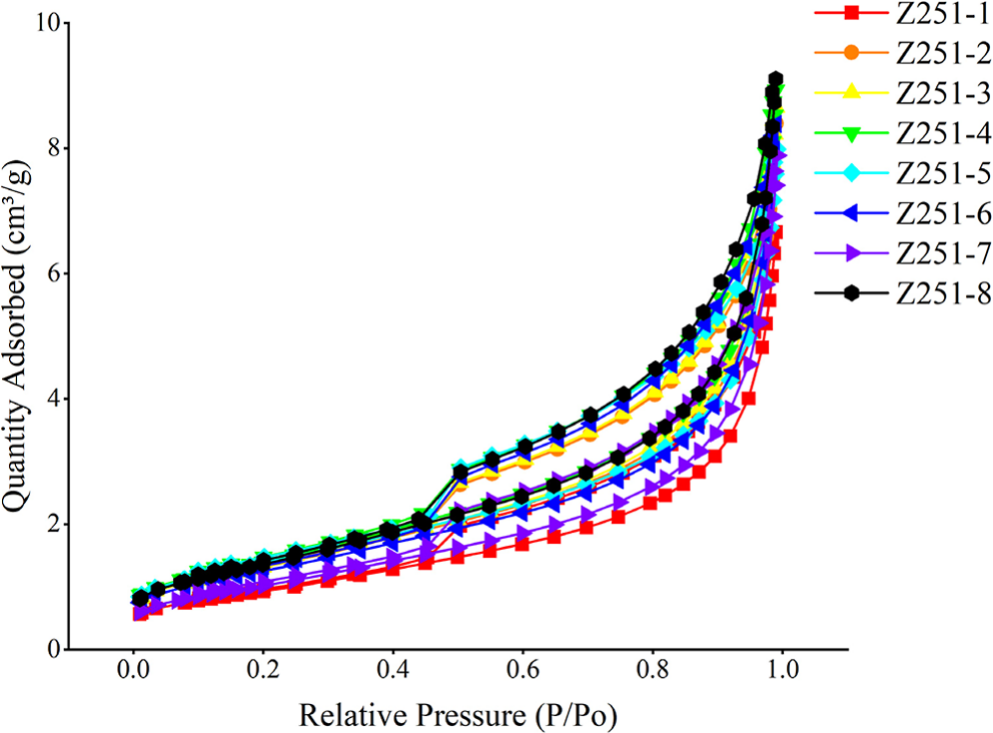
N_2_ and desorption isotherms for Z251.

Regarding pore size classification, the IUPAC scheme is commonly adopted, categorizing pores into micropores (less than 2 nm), mesopores (2–50 nm), and macropores (more than 50 nm). The N_2_ isotherm experiment is particularly precise for the characterization of mesopores.^61^ Pore size distribution characteristics are typically characterized using differential, incremental, and cumulative distribution curves, each providing distinct information about the range, volume, and change in pore size. This study employs the incremental distribution curve, with the abscissa representing the range of pore sizes and the ordinate representing the incremental volume of pores (Figure 4). Previous research has indicated that this curve exhibits normal distribution characteristics; therefore, the peak height of the ordinate can represent the concentration of the pore size distribution, characterizing the principal range of pore sizes.^7–9^ Based on the hysteresis loop classification, these pores represent a transitional type between H3 and H4, including morphologies of parallel plate-like and unilateral slit pores.

**Figure 4:**
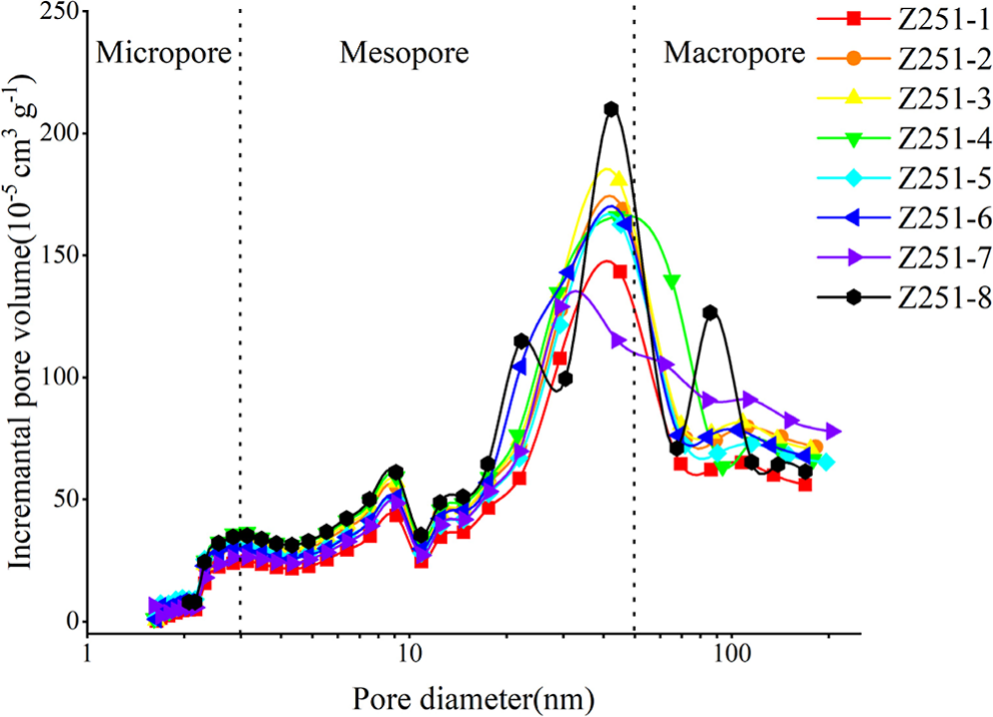
MicroPV characteristics of Z251.

The adsorption characteristics of the eight samples exhibit similar trends, where the adsorbed volume within each relative pressure range indicates the proportional distribution of pore sizes within that range. The relative pressure interval from 0 to 0.4 *P*/*P*_0_ approximately matches a pore diameter range of 0–2 nm. Within this pressure range, the adsorption isotherm changes gradually, displaying a slight convex upward trend indicative of the presence of a significant quantity of micropores, with a very narrow hysteresis loop. As the relative pressure increases from 0.4 to 0.8, corresponding to the pore diameter range of 2–50 nm, the adsorption isotherm rises progressively, signifying an increase in adsorbed volume that reflects a transition from smaller to larger pores. At a relative pressure between 0.8 to 1, representing a pore diameter of 50–200 nm, the adsorption isotherm sharply increases, and the curve exhibits a pronounced concave feature with an increased hysteresis loop width. This denotes the shift from monolayer to multilayer adsorption within shale pores, indicating a transition from micropores to macropores, consistent with the isotherm characteristics of terrestrial shales.^40^ Regarding the desorption isotherms, they predominantly exhibit a rapid decline at a relative pressure of 0.8, with a steep slope. When the relative pressure falls below 0.8, the desorption curves slightly decline with a smaller slope. At a relative pressure of 0.5, after a rapid decrease, the desorption curve shows a noticeable steep drop, ultimately closing or diverging with a smaller maximum adsorption capacity.

Generally, macropores provide PV, while micropores contribute to the SSA. Based on the peak variation patterns in pore size distribution, the shale samples are categorized into two types. For pore diameters less than 2 nm, all samples exhibited no significant changes in PV. However, for pore diameters between 2 and 50 nm, sample number 8 displayed two distinct peaks, whereas the remaining samples exhibited only a single peak. For pore diameters larger than 50 nm, sample number 8 showed a single peak, while no peaks were observed in the other samples, suggesting a relatively concentrated mesopore presence in most samples, whereas the distribution in sample 8 was broader, possibly due to experimental errors­(Figure 5). The SSAs of the eight samples ranged from 3.42 to 5.13 m^2^/g, with PVs from 10.9 to 15 cm^3^/kg. In comparison, terrestrial shales exhibited SSAs between 1.1 and 1.9 m^2^/g and PVs from 6.9 to 10.9 cm^3^/kg, slightly higher than those of lacustrine shales in China.^9,10,57,62^ The distribution of mesopores dominated the PV measurements, accounting for 7.7 to 10.85 cm^3^/kg, followed by macropores at 3.07 to 4.47 cm^3^/kg, and micropores being the least at 0.12 to 0.34 cm^3^/kg. This distribution is likely associated with the primary use of low-temperature N_2_ experiments characterizing mesopores.^63^

**Figure 5:**
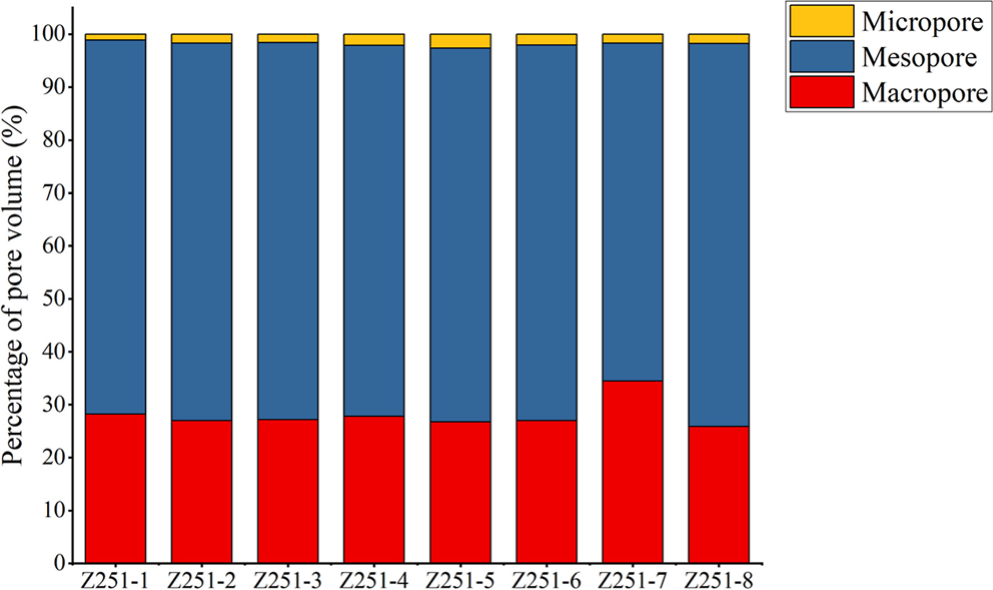
Histogram of the percentage of PV.

#### 4.2.2 HPMI

HPMI is one of the most commonly employed methodologies in reservoir studies. Mercury, as a nonwetting phase, requires an external force to penetrate porosities. Due to the influence of the mercury molecule radius, HPMI methodology exhibits specific applicability. For pores of smaller diameters, particularly at the nanoscale, extremely high external pressures are necessary for mercury to infiltrate these pores. However, excessive pressures may compromise the original structure of the pores, creating new fractures and rendering the measurement results unexpectedly unreliable. Consequently, HPMI is frequently utilized to characterize the macropores within shale formations.^64^

Figure 6 illustrates the capillary pressure curves for eight samples. The mercury intrusion and extrusion curves generated by the HPMI method reflect the extent of development and connectivity of pores within different ranges. At low pressures, the mercury entry volume increases rapidly, indicated by the steepest slope of the curve, suggesting the presence of numerous micrometer-sized pores in the shale samples. As the pressure reaches an intermediate range, the slope of the curve becomes more gradual, and the rate of mercury intrusion slows down. Under high-pressure conditions, as the pressure continues to rise, the volume of mercury entering does not increase further; at this stage, the mercury intrusion curve becomes linear, with a slope approaching zero. This indicates that pore development within this range is minimal. When the pressure is sufficiently high, the mercury volume starts to increase further until it reaches its maximum value, indicating the presence of smaller nanoscale pores within the samples. The degree of closure in the mercury migration curves suggests that the majority of the shale samples have larger openings, indicating a generally poor connectivity of the pores. Samples 2, 4, and 8 exhibit more open and better-connected porosity structures.

**Figure 6:**
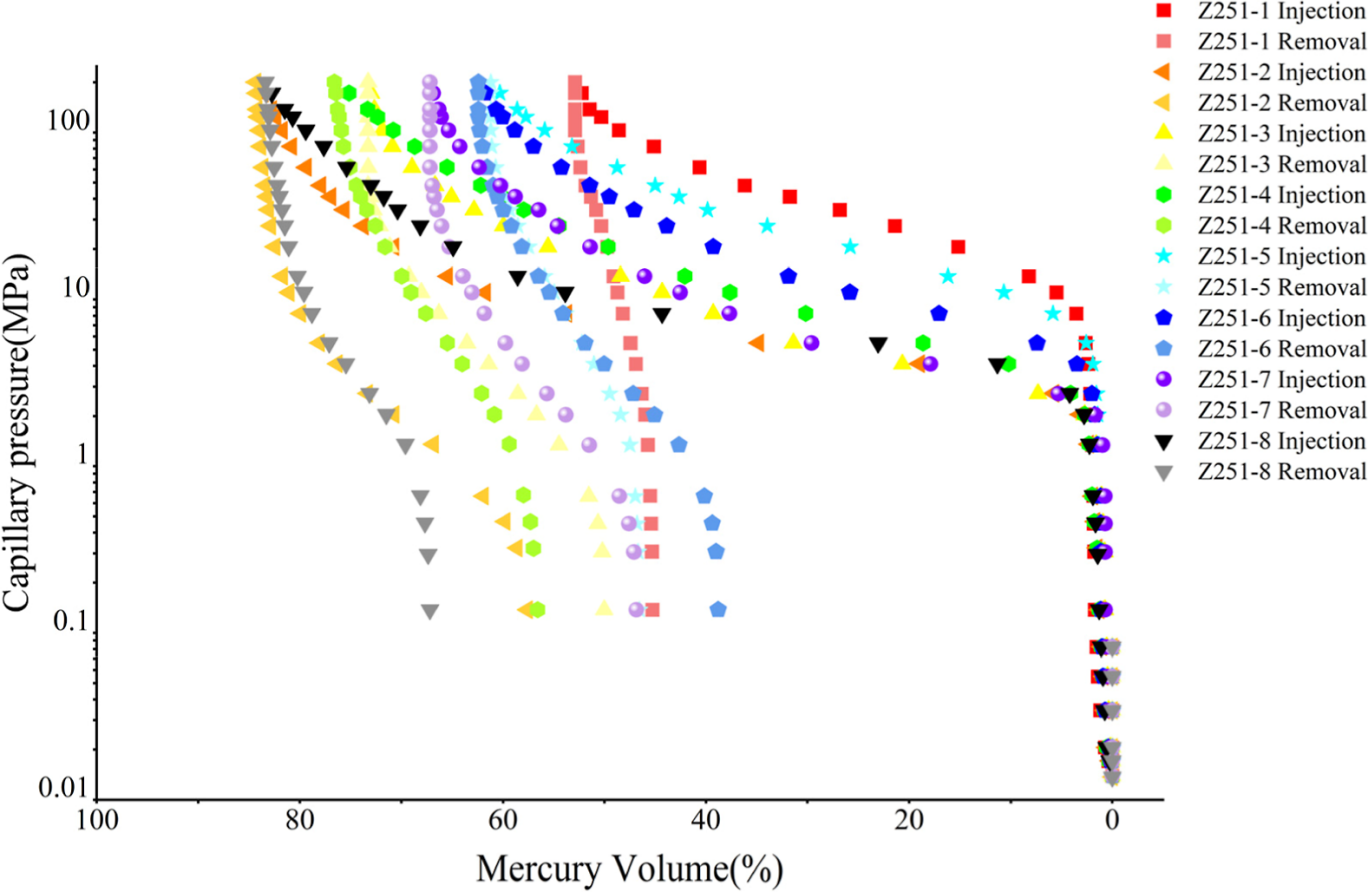
Connection between capillary force and mercury volume/saturation (injection and removal).

The pore radius at various pressure points can be determined by measuring the pressure exerted during the mercury intrusion procedure and using the Washburn equation, with the corresponding mercury volume representing the PV. Figure 7 displays notable differences in the overall pore size distribution among shale samples. Samples 1 and 5 exhibit peak pore sizes around 0.025 μm, whereas samples 2 and 8 show distributions around 0.1 μm, indicating that the latter two samples possess a larger distribution of pore sizes. The vertical axis, which denotes the frequency of pore distribution, indicates the likelihood of pore occurrence; a lower peak suggests fewer pores. When comparing samples 4 and 6 with samples 2 and 8, it is evident that despite having similar pore size distributions around 0.1 μm, samples 4 and 6 contain fewer pores. Although samples 3 and 7 show a concentrated distribution of pores between 0.15 and 0.2 μm, they have significantly fewer pores compared to samples 2 and 8. Besides macropores, shale samples 1 and 5 also exhibit the development of mesopores. Despite originating from the same geological formation, the extent of pore development varies greatly among the samples. This variation may be attributed to differences in shale mineral composition, OM richness, and burial depth under different depositional microfacies, leading to varying degrees of thermal evolution.^3,16,19,22^ Figure 8 illustrates the contribution of various pore size ranges to permeability across the samples, with characteristics similar to those observed in Figure 7. Samples 1 and 5 show a lower distribution of pore sizes. Samples 3 and 7 exhibit larger pore sizes. Notably, aside from sample 6, which has a permeability contribution rate of 30%, the influence of pore size on permeability for the other samples is approximately similar, ranging from 37.5% to 45%.

**Figure 7:**
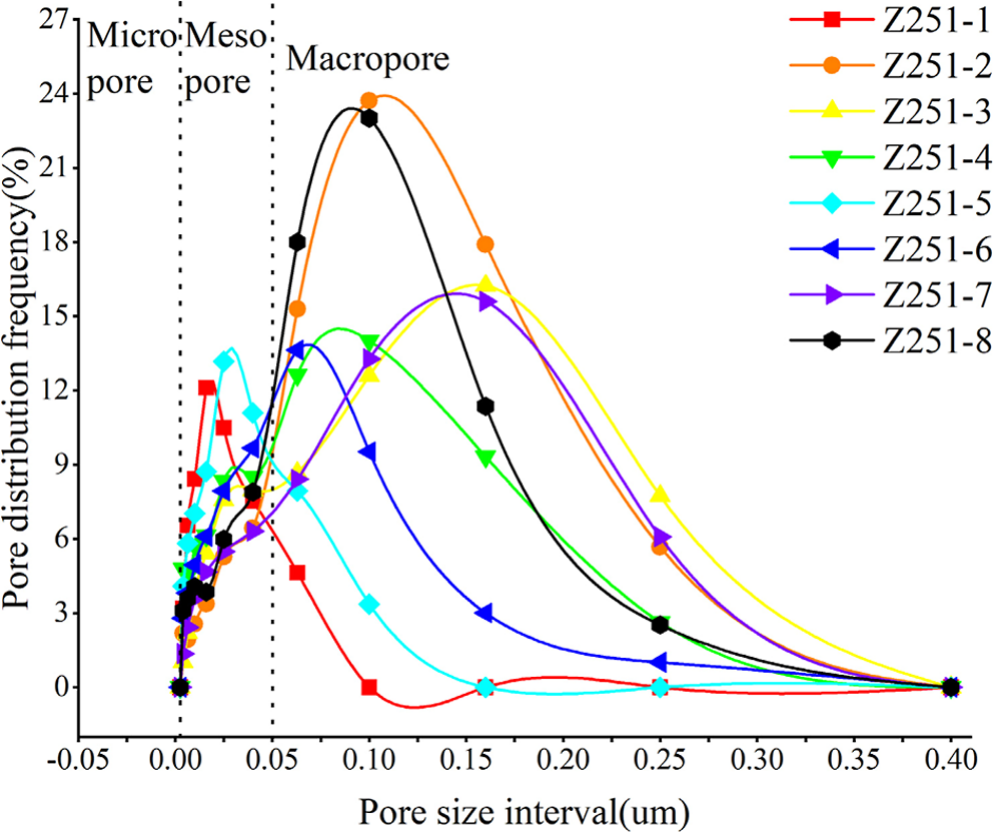
Pore distribution frequency under varying pore size intervals.

**Figure 8:**
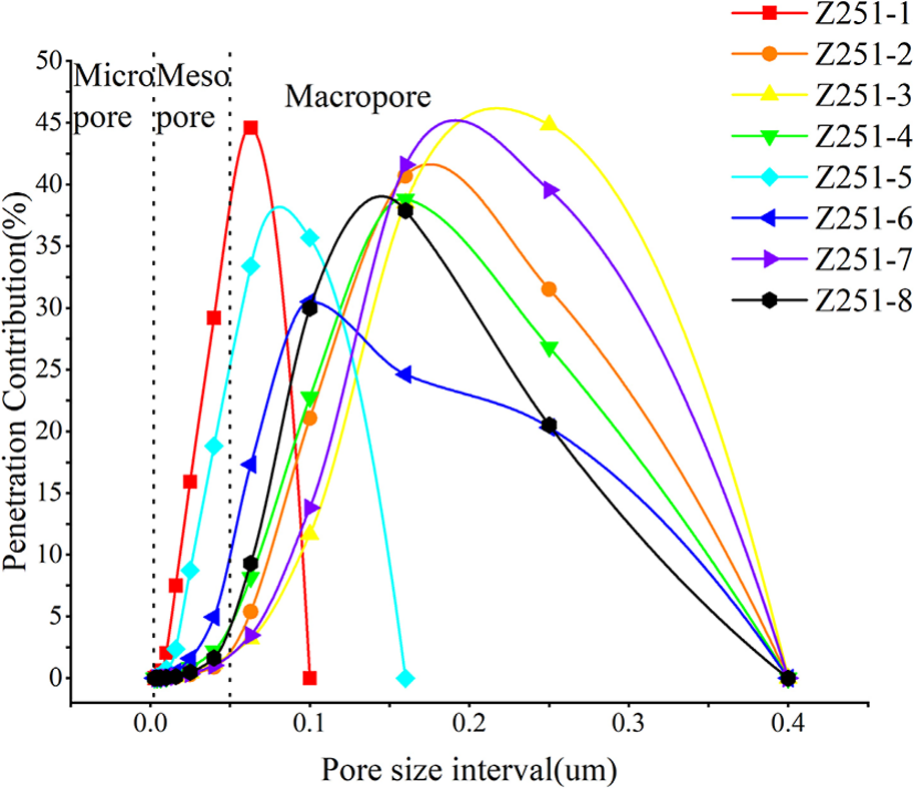
Permeability contribution rate under varying pore size intervals.

Table 4 presents the data on average pore radius, mercury extrusion efficiency, maximum mercury saturation, and displacement pressure for eight samples. Samples 1, 3, and 7 exhibited lower average pore radii. The mercury extrusion efficiency was lower for samples 1 and 8, whereas samples 1 and 5 demonstrated low maximum mercury saturations and high displacement pressures. Mercury extrusion efficiency is indicative of the pore structure connectivity within the samples; a higher efficiency suggests better interconnectivity. The parameters of average pore radius, maximum mercury saturation, and displacement pressure collectively facilitate an assessment of the sample’s pore characteristics. A smaller average pore radius correlates with lower maximum mercury saturation and higher displacement pressure, indicating that mercury penetration into the sample is more challenging when the pores are smaller. These findings are consistent with the conclusions drawn from Figures 7 and 8,.^64^

**Table 4:** Pore Parameters of High-Pressure Mercury Injection in Well Z251

sample ID	depth (m)	TOC (%)	average pore radius (um)	mercury removal efficiency (%)	maximum mercury saturation (%)	exhaust pressure (Mpa)
Z251-1	1612.15	2.90	0.024	14.40	52.91	8.25
Z251-2	1617.88	1.82	0.11	31.68	84.35	2.05
Z251-3	1630.33	2.19	0.033	31.74	73.28	2.04
Z251-4	1630.86	1.62	0.061	26.12	76.62	2.74
Z251-5	1636.22	2.63	0.068	23.92	61.20	5.49
Z251-6	1637.85	2.17	0.11	37.86	62.44	2.73
Z251-7	1656.11	2.27	0.079	30.27	67.20	2.04
Z251-8	1672.02	2.08	0.11	19.39	83.37	2.74

### 4.3 Pore Morphology Characteristics

Shale reservoirs are typically characterized by low porosity and permeability, along with complex structures, making the AIP-FESEM an effective method for direct visualization of the pore structures.^11,65^ Figure 9 compiles images from standard thin sections, cast thin sections, and AIP-FESEM photographs. The majority of the samples exhibit development of felsic (quartz and feldspar) intergranular pores with fewer instances of feldspar dissolution pores, and occasional microfractures observed. Under microscopic examination in Figure 9A,B, a range from very fine to fine sandy textures can be observed, covering lithic fragments, feldspar, and quartz, with minor inclusions of mica and flint; OM and minor lithic debris are also present. The feldspar observed includes both potassium feldspar and plagioclase, with some feldspars displaying alteration, predominantly into Illite. The lithic fragments are mainly acidic volcanic rocks, with minor chert, quartzite, and mudstone fragments; mica exhibits a flattened and bent morphology, primarily distributed between grains. Intergranular spaces contain minerals such as dolomite, minor mudstone, calcite, siderite, and dolomite infillings, with mudstone showing illitization and carbonates containing clastic replacements. Pore development is generally limited. In Figure 9C, a similar very fine to fine sandy texture is observed, differing from Figure 9B in that the sample exhibits poor pore development with intergranular dolomite infillings. In Figure 9D, a carbonate very fine to fine sandy texture is visible, similar to Figure 9C, but with extremely poor pore development; Figure 9E shows similar textural features with poor pore development.

**Figure 9:**
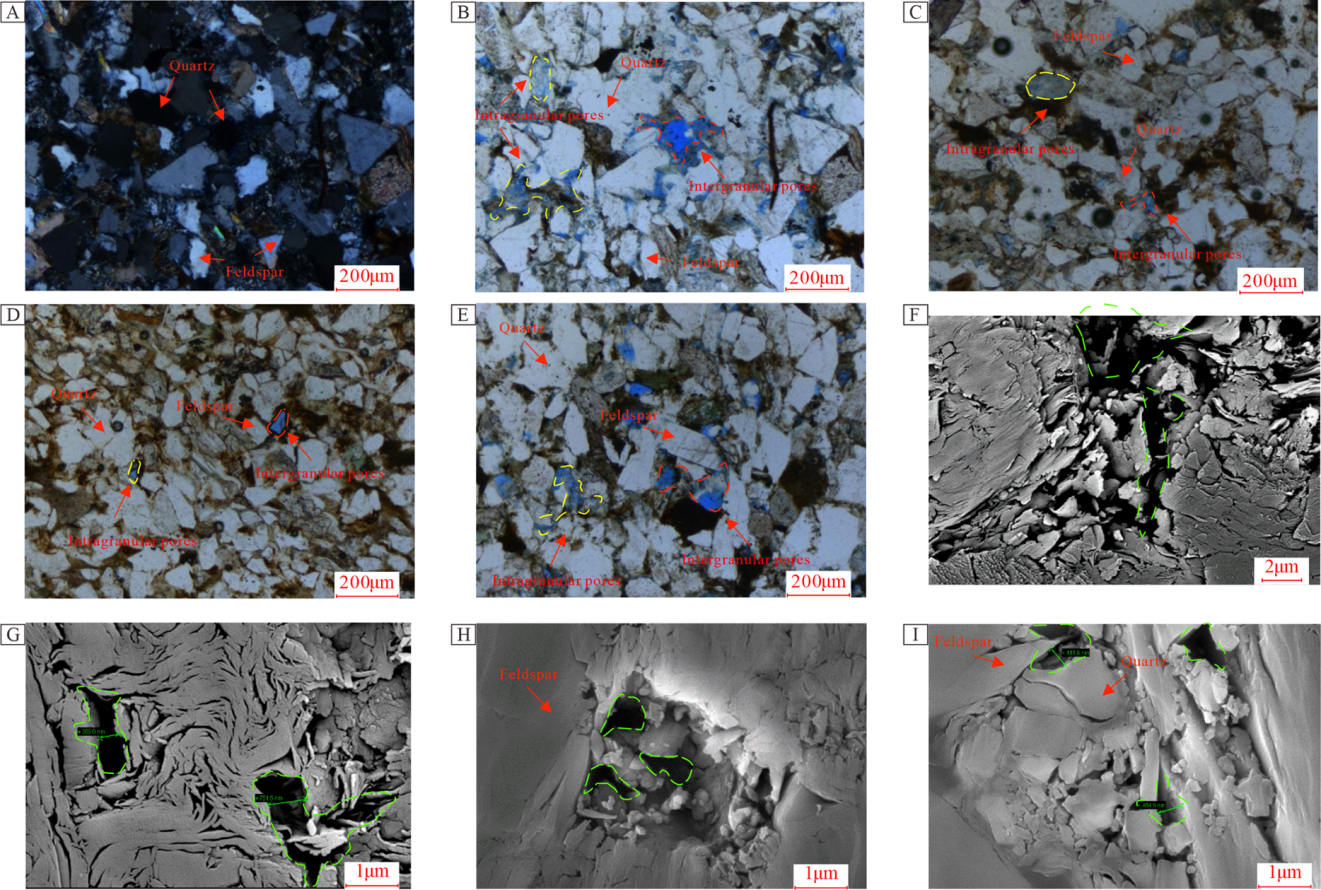
Images of standard thin sections, cast thin sections, and AIP-FESEM for samples. Panel A: Sample Z251-1 at a depth of 1612.15 m, comprising quartz, feldspar, and rock fragments; Panel B: Sample Z251-1 at the same depth, featuring intergranular pores and minor feldspar dissolution pores with moderate pore development; Panel C: Sample Z251-3 at a depth of 1630.33 m, characterized by intergranular pores and few feldspar dissolution pores, showing poor pore development; Panel D: Sample Z251-6 at a depth of 1637.85 m, displaying intergranular pores and feldspar dissolution pores, with poor pore development; Panel E: Sample Z251-7 at a depth of 1656.11 m, exhibiting intergranular pores and feldspar dissolution pores, indicating poor pore development; Panel F: Sample Z251-2 at a depth of 1617.88 m, demonstrating intragranular porosity due to illitization, with pore spaces ranging from 1 to 2 μm; Panel G: Sample Z251-4 at a depth of 1630.86 m, showing intragranular porosity with Illite and montmorillonite clay mineralization, and pore spaces between 30 and 80 nm; Panel H: Sample Z251-5 at a depth of 1636.22 m, illustrating intergranular pores in feldspar, with pore spaces (20–70 nm); Panel I: Sample Z251-6 at a depth of 1637.85 m, displaying intergranular pores in feldspar, with pore spaces between 40 and 50 nm. (Line colors: red for intergranular pores; yellow for intragranular dissolution pores; green for the range of porosity spaces.).

Figure 9F reveals felsic intergranular pores with pore spaces ranging from 1 to 2 μm. Some regions display intragranular pores formed by feldspar dissolution. In Figure 9G, it is evident that felsic intergranular pores are filled with CMs, with pore spaces ranging from 20 to 80 nm. Figure 9H displays intergranular pores filled with calcite, with pore spaces between 20 and 70 nm. Lastly, Figure 9I illustrates felsic intergranular pores filled with minerals such as calcite and dolomite, with pore spaces (40 to 50 nm).

In heterogeneous samples, different minerals exhibit distinct physical properties. CMs (e.g., montmorillonite and Illite) are strongly hydrophilic with high SSAs, whereas quartz and feldspar are hydrophobic with relatively low surface energy. In N_2_ adsorption tests, pores dominated by quartz tend to have larger contact angles, leading to underestimated adsorption volumes and, consequently, underestimated pore radii.^66,67^ By contrast, clay-dominated pores exhibit smaller contact angles, which can result in overestimated adsorption volumes; during calculations, this overestimation is often misinterpreted as “larger pore radii,” even when the actual pore sizes are small.^68^ In HPMI tests, pores lined with calcite require higher mercury injection pressures, producing calculated pore radii smaller than their actual sizes, whereas pores lined with clay (which has lower surface tension) permit mercury intrusion at lower pressures, leading to overestimated radii.^69^ Mixed clay-quartz slit-shaped pores also tend to yield overestimated radii, while quartz-dominated cylindrical pores are calculated more accurately, as the actual pore radius is smaller than the theoretical value. Moreover, the dissolution of unstable minerals such as feldspar and calcite often generates ink-bottle-shaped pores with narrow throats and wide bodies.^43^ In such cases, HPMI measures only the throat radius (as mercury must first pass through the throat), whereas gas adsorption methods measure the body radius during desorption. This discrepancy can produce differences of one to 2 orders of magnitude in calculated pore radii.^43,70^ To address this, the present study combines HPMI and N_2_ adsorption, targeting macropores^64^ and mesopores,^61^ respectively, and integrates XRD to determine mineral composition. This multimethod approach provides complementary perspectives, improves measurement accuracy, and minimizes errors when evaluating factors that influence pore structures.

## 5 Discussion

### 5.1 Characteristics of Pore Development

The microscale and nanoscale pore morphology of shale reservoirs exhibits a diverse array, and shale exhibits a complex and highly heterogeneous structure. This heterogeneity significantly impacts the reservoir’s storage and connectivity capabilities.^5^ A detailed characterization of the development and distribution of pore spaces in eight samples was conducted using liquid N_2_ and HPMI experiments. Samples 3, 4, and 8 displayed a higher total PV compared to samples 1, 2, 5, 6, and 7, and also exhibited larger pore radii, indicating that a larger PV, particularly in mesopores, enhances the storage capacity and performance of shale (Table 3). Samples 4 and 5 had a higher SSA than samples 2, 3, 6, and 7, but exhibited lower mercury recovery efficiency, suggesting that while a larger SSA provides more adsorption sites for hydrocarbons, making it relatively more favorable for hydrocarbons, it also indicates a more complex and tortuous pore structure^33^ (Tables 3 and 4).

OM pores are formed within OM during thermal maturation and hydrocarbon generation processes and are predominantly developed within the organic material itself. These pores are generally spherical, elliptical, or irregularly shaped and are sporadically distributed (Figure 9B–D). A significant presence of intergranular pores, characterized by dissolution features and more regular pore morphologies, was observed in all eight shale samples. Typically, these regular pores are predominantly inorganic and are secondary intergranular dissolution pores formed through later-stage dissolution processes, primarily appearing in isolated forms with poor connectivity.^3,19,22,41,65^ However, the proportion of intragranular dissolution pores observed in thin sections was minimal, contributing insignificantly to the overall pore space.

### 5.2 Factors Controlling Pore Development

Generally, shale’s evolution involves sedimentation, compaction, diagenesis, and thermal maturation of OM leading to hydrocarbon generation. Multiple factors affect pore space development.^65^

#### 5.2.1 OM Content

The influence of organic carbon content on PV in eight samples demonstrated a negative correlation, as depicted in Figure 10. Some scholars assert that the presence of OM promotes pore development in shale reservoirs, and its contribution becomes apparent only once a certain threshold of abundance is reached.^63^ In images 9E-I, pores associated with OM (OM pores) are minimally present and nearly imperceptible. The adverse effects of OM content on mesopores and macropores indicate that an increase in organic carbon suppresses the PV and SSA of macropores and mesopores (Figure 10A). This finding further corroborates the low maturity and poor abundance of continental shale in the Longdong area. This conclusion aligns with characteristics of pore development observed in Permian shales from other basins.^65,71^ The lithofacies of the eight samples are characterized as silty shale, where OM pores are underdeveloped and OM pores’ volume is minimal. Inorganic minerals’ pores remain the predominant pore type. Thus, the content of OM exerts an inhibitory effect on pore development.

**Figure 10:**
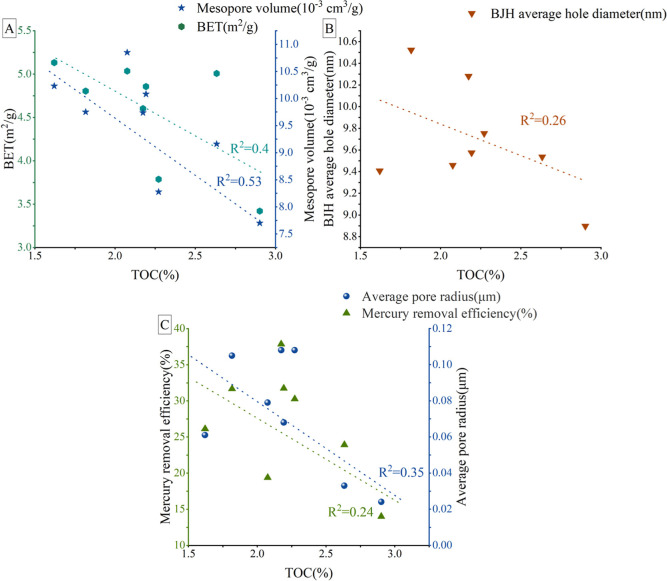
Connection between OM content and mesopores/macropores: (A): connection between OM content and BET/mesopore volume; (B): connection between OM content and BJH average hole diameter; (C): connection between OM content, mercury removal efficiency, and average macropore radius).

#### 5.2.2 The Rock Composition

Mineral constituents form the fundamental framework of rock structures, and each mineral exhibits unique physicochemical properties. OM undergoes various changes during diagenesis and thermal evolution, which differently impacts the development of pores in mudstone.^63^ The findings in Section 5.2.1 infer that the volume of mesopores and macropores is associated with inorganic minerals, leading to a reduction in inorganic mineral content with the increasing OM pore content.

The Ordos Basin primarily consists of terrestrial and transitional marine shale formations as its shale reservoirs. Within this context, a study of eight samples revealed that inorganic pores predominantly characterize these reservoirs, with siliceous minerals serving as the principal mineral constituents. There exists a positive connection between siliceous minerals’ content and the volume of mesopores and macropores, as evidenced by Figure 11. Mineral composition has complicated effects on porosity development within shale reservoirs. Notably, brittle minerals, primarily felsic, positively influence the development of porosity, especially enhancing the formation of mesopores and macropores.^63^ Conversely, the effect exerted by carbonate rock minerals on the pore characteristics such as PV, SSA, and pore radius of mesopores and macropores is not significant, as shown in Figure 12. This minimal effect can be attributed to the low proportion (1–5%) of carbonate minerals relative to felsic, which plays a dominant role in the interstitial spaces between particles, thereby having a negligible effect on mesopores and macropores and potentially inhibiting the connectivity of macropores, as indicated in Figure 12C. Furthermore, an inverse association exists between the content of CMs and the volume of mesopores and macropores, as depicted in Figure 13. The proportion of CMs ranges from 6 to 21%, with a particularly strong negative correlation with mesopores’ PV and SSA. This adverse effect is due to the positive role of felsic in pore formation, where fine-grained clay transported and deposited along with coarser detritus during sedimentation obstructs the intergranular pores of felsic, particularly affecting the mesopore volume and SSA, as shown in Figure 13. In summary, while felsic minerals promote porosity development in the shale reservoir, CMs tend to inhibit this development. The impact of carbonate minerals on porosity is minimal.

**Figure 11:**
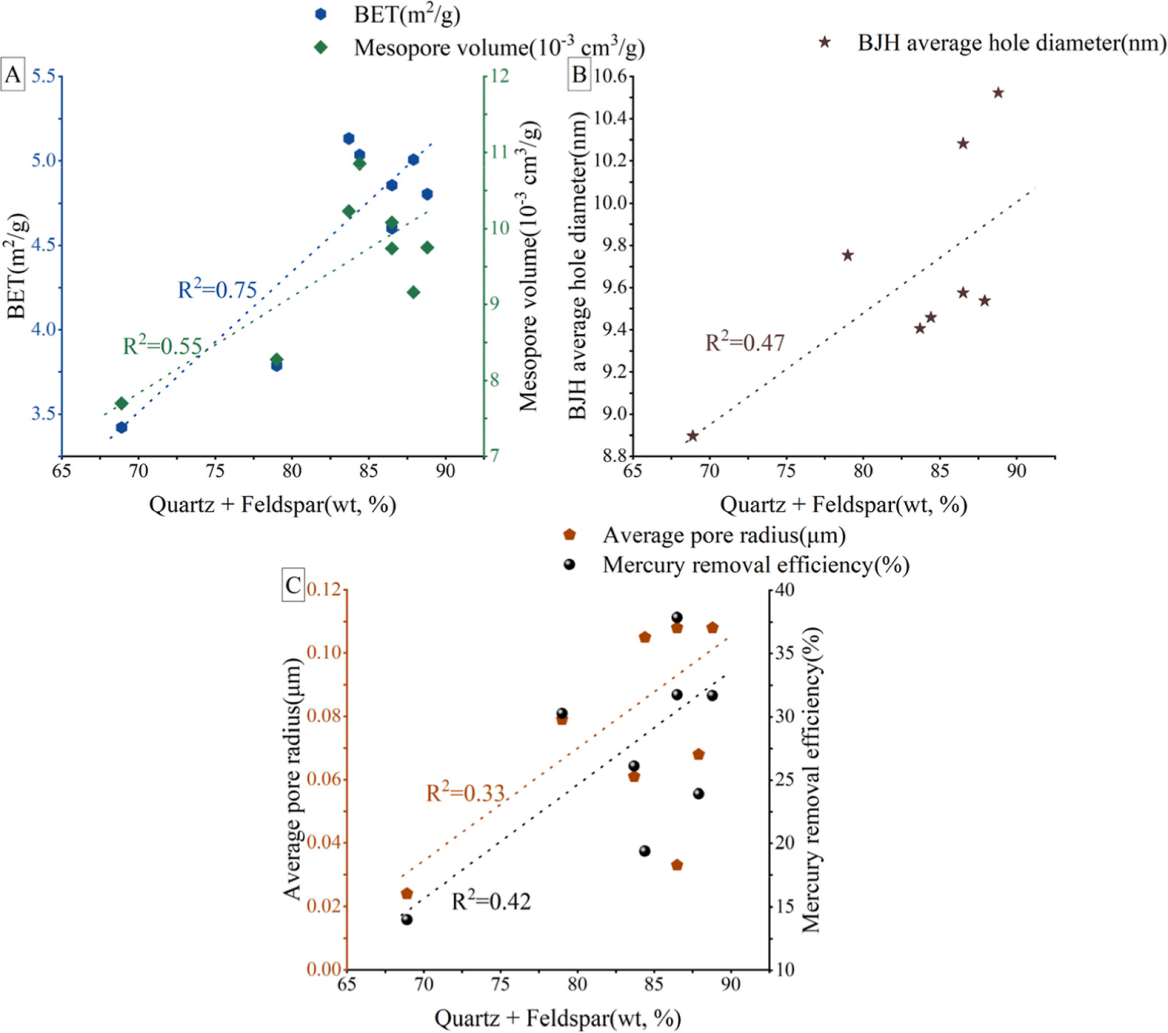
Connection between Quartz + Feldspar and mesopores/macropores: (A): connection between Quartz + Feldspar content and BET/mesopore volume; (B): connection between Quartz + Feldspar and BJH average hole diameter; (C): connection between Quartz + Feldspar, mercury removal efficiency, and average macropore radius).

**Figure 12:**
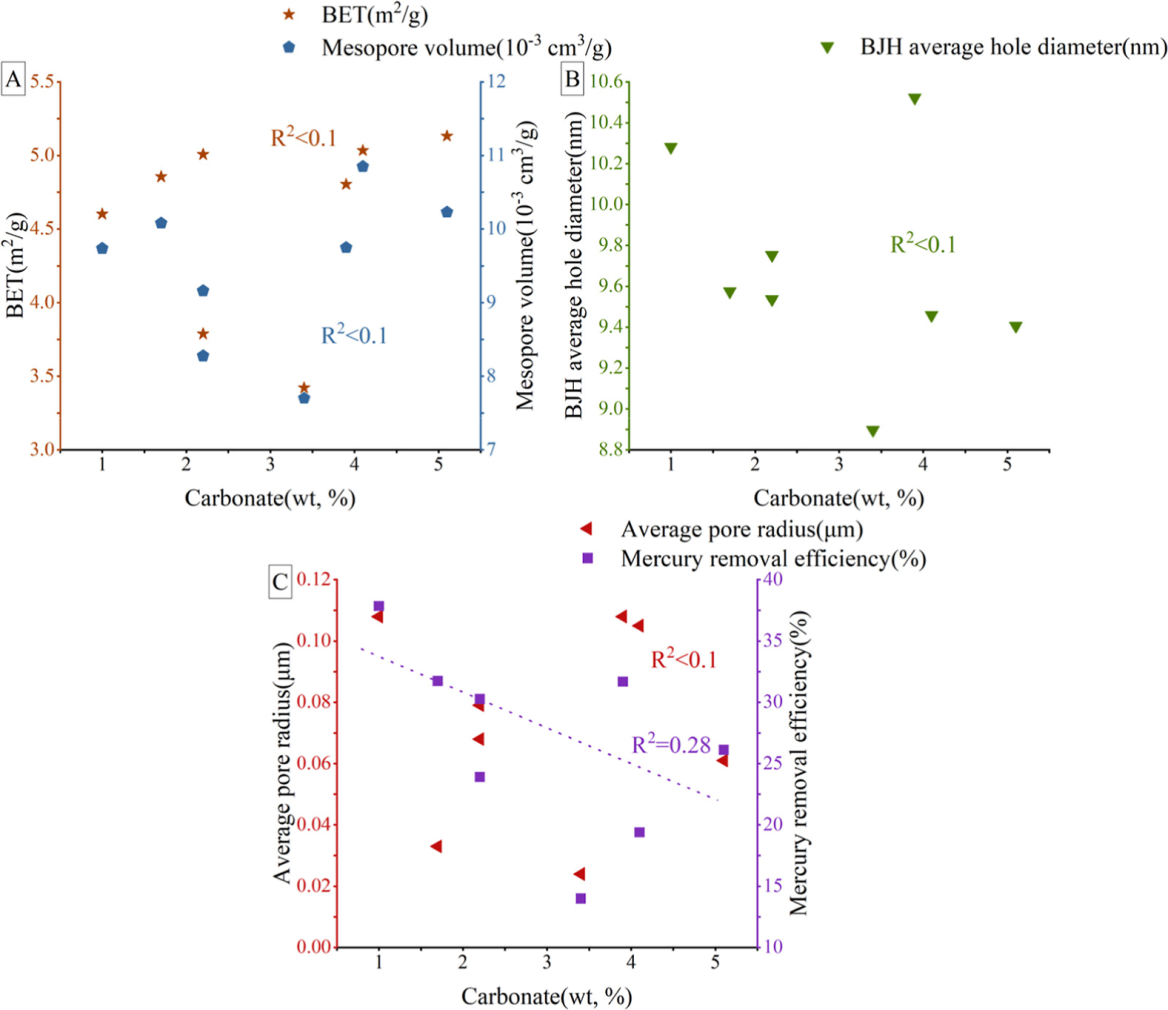
Connection between Carbonate and mesopores/macropores: (A): connection between Carbonate content and BET/mesopore volume; (B): connection between Carbonate and BJH average hole diameter; (C): connection between Carbonate, mercury removal efficiency, and average macropore radius).

**Figure 13:**
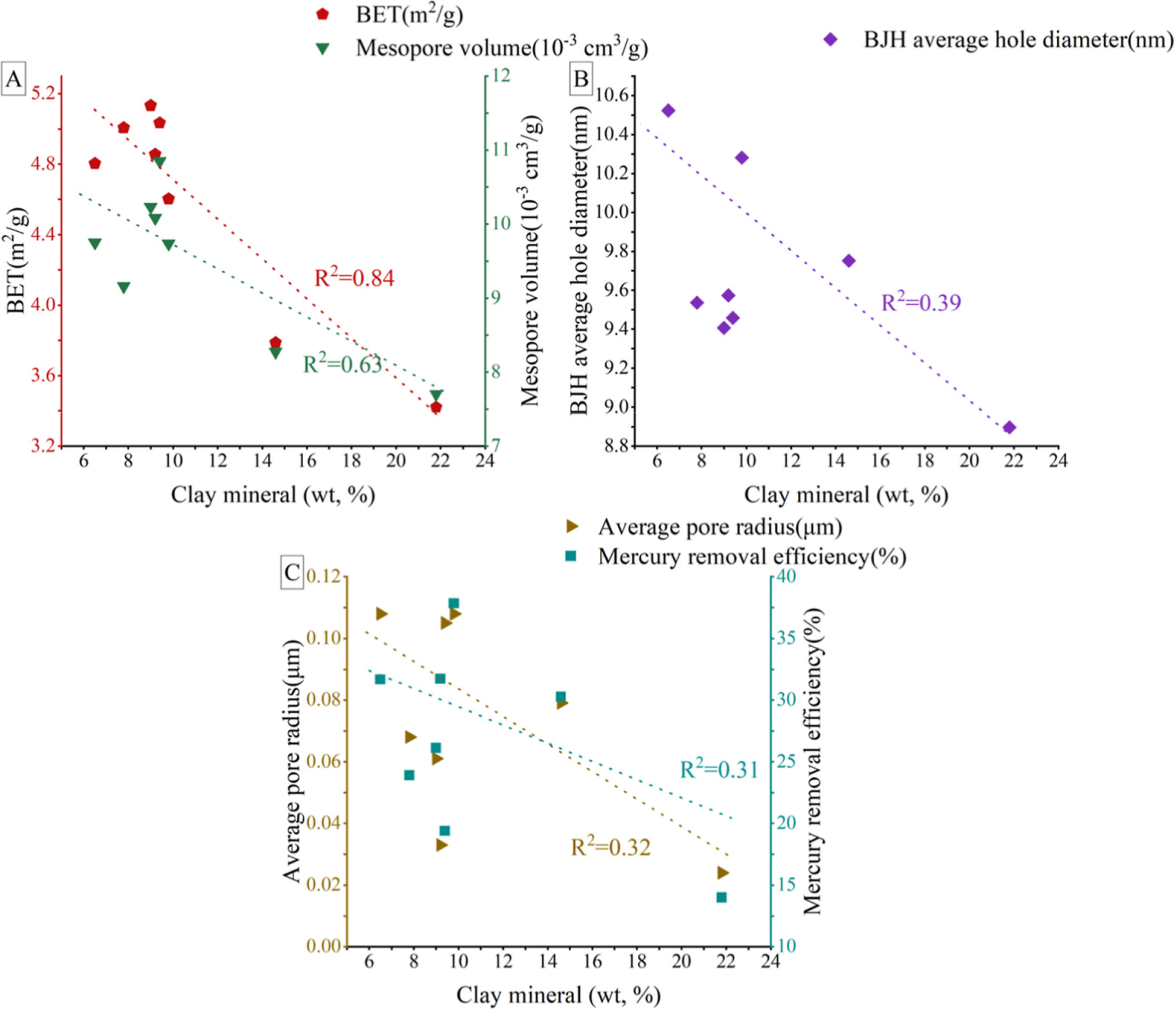
Connection between CM and mesopores/macropores: (A): connection between CM content and BET/mesopore volume; (B): connection between CM and BJH average hole diameter; (C): connection between CM, mercury removal efficiency, and average macropore radius).

## 6 Conclusions

Based on the analysis of rock mineral compositions, HPMI, and N_2_ experiments, in conjunction with observations from thin sections, cast thin sections, and AIP-FESEM of eight samples from the Chang7 segment in the Longdong area, we draw the conclusions below.

(1) The pore radius of silty shale determines the PV of the shale reservoir, especially the mesopore spaces. A larger pore radius correlates with an increased storage capacity. The SSA governs the reservoir’s connectivity, thereby influencing the pore structure’s complexity. A larger SSA results in more complex pore configurations, facilitating enhanced hydrocarbon adsorption.
(2) Organic carbon content impacts pore development in silty shales, particularly affecting the PV of mesopores and macropores. As organic carbon content increases, PV decreases, attributed to the maturity of the OM not reaching the threshold necessary for hydrocarbon generation. This results in a lower proportion of organic pores compared to inorganic mesopores and macropores.
(3) Siliceous minerals in shale positively influence the PV, with both macropore and mesopore volumes, as well as the SSA, increasing with the content of siliceous minerals. A notable proportion of felsic intergranular pores contributes to the advantageous pore spaces in shale oil reservoirs.
(4) CMs, with increasing content, suppress the PV and SSA of both macropores and mesopores. Carbonate rock minerals, however, have a minimal effect on these pore sizes’ PV and SSA. Future research should focus on how CMs influence pore structures. The filling of CMs reduces the pore space in felsic intergranular pores, whereas the presence of carbonate rock minerals, being minimal in the rock composition, has a negligible impact on porosity. These results not only reveal the primary controlling factors of pore development in the Chang 7 Member shale reservoirs of the Longdong area but also offer important implications for shale oil exploration and development. Siliceous-rich, clay-poor silty shales should be prioritized as exploration targets, whereas intervals with relatively high OM content that have not yet reached the hydrocarbon generation threshold require integrated evaluation alongside their thermal evolution stage to avoid overestimating reservoir potential.
